# Towards targeted colorectal cancer biopsy based on tissue morphology assessment by compression optical coherence elastography

**DOI:** 10.3389/fonc.2023.1121838

**Published:** 2023-03-27

**Authors:** Anton A. Plekhanov, Marina A. Sirotkina, Ekaterina V. Gubarkova, Elena B. Kiseleva, Alexander A. Sovetsky, Maria M. Karabut, Vladimir E. Zagainov, Sergey S. Kuznetsov, Anna V. Maslennikova, Elena V. Zagaynova, Vladimir Y. Zaitsev, Natalia D. Gladkova

**Affiliations:** ^1^ Institute of Experimental Oncology and Biomedical Technologies, Privolzhsky Research Medical University, Nizhny Novgorod, Russia; ^2^ Laboratory of Wave Methods for Studying Structurally Inhomogeneous Media, Institute of Applied Physics Russian Academy of Sciences, Nizhny Novgorod, Russia; ^3^ Department of Faculty Surgery and Transplantation, Privolzhsky Research Medical University, Nizhny Novgorod, Russia; ^4^ Department of Pathology, Nizhny Novgorod Regional Oncologic Hospital, Nizhny Novgorod, Russia; ^5^ Department of Oncology, Radiation Therapy and Radiation Diagnostics, Privolzhsky Research Medical University, Nizhny Novgorod, Russia; ^6^ Lobachevsky State University of Nizhny Novgorod, Nizhny Novgorod, Russia

**Keywords:** compression optical coherence elastography (C-OCE), optical biopsy, morphology assessment, colon tissue, colorectal cancer

## Abstract

Identifying the precise topography of cancer for targeted biopsy in colonoscopic examination is a challenge in current diagnostic practice. For the first time we demonstrate the use of compression optical coherence elastography (C-OCE) technology as a new functional OCT modality for differentiating between cancerous and non-cancerous tissues in colon and detecting their morphological features on the basis of measurement of tissue elastic properties. The method uses pre-determined stiffness values (Young’s modulus) to distinguish between different morphological structures of normal (mucosa and submucosa), benign tumor (adenoma) and malignant tumor tissue (including cancer cells, gland-like structures, cribriform gland-like structures, stromal fibers, extracellular mucin). After analyzing in excess of fifty tissue samples, a threshold stiffness value of 520 kPa was suggested above which areas of colorectal cancer were detected invariably. A high Pearson correlation (r =0.98; p <0.05), and a negligible bias (0.22) by good agreement of the segmentation results of C-OCE and histological (reference standard) images was demonstrated, indicating the efficiency of C-OCE to identify the precise localization of colorectal cancer and the possibility to perform targeted biopsy. Furthermore, we demonstrated the ability of C-OCE to differentiate morphological subtypes of colorectal cancer – low-grade and high-grade colorectal adenocarcinomas, mucinous adenocarcinoma, and cribriform patterns. The obtained *ex vivo* results highlight prospects of C-OCE for high-level colon malignancy detection. The future endoscopic use of C-OCE will allow targeted biopsy sampling and simultaneous rapid analysis of the heterogeneous morphology of colon tumors.

## 1 Introduction

Colorectal cancer accounts for 8-12% of oncological diseases globally (1). The risk of developing colorectal cancer in young people is fairly low. However, it rises sharply after the age of 50, being predominantly associated with cancer development from benign mucosal hyperplasia, malignancy of which occurs on average in 10-15 years (2). Importantly, mortality from colorectal cancer is low if the disease is detected in early stages and treated appropriately.

Routine clinical examinations of patients with suspected colorectal cancer include history taking, fecal occult blood test, digital rectal examination, colonoscopy with possible contrast enhancement and parallel biopsy (3). Instrumental diagnostic methods are frequently used preoperatively for patient screening and staging of colorectal cancer. Computed tomography, colonography, positron emission tomography and magnetic resonance imaging (MRI) provide anatomic information about tumors and determine regional and distant foci of metastasis (prevalence of colorectal cancer progression) (4). The results of these methods are also essential in assessing a prognosis and developing a treatment strategy (5). Furthermore, these technologies can be used to morphologically characterize the affected colon wall (6) within their resolution limitations. Notably, morphological analysis of colorectal cancer along with screening and staging is the most important factor in selecting therapeutic and surgical tactics.

To obtain information about morphological structure of colorectal cancer and establish cancer subtype a biopsy tissue sample during colonoscopy should be taken and a consequent histological examination should be performed. The pathologist identifies the colorectal cancer subtype based on the combination of morphological and molecular features. In recent studies, the molecular status and response to therapy of most colorectal cancer morphological subtypes becomes the main factor in the pathology diagnosis of the tumor in its most malignant area (7, 8). In light of high morphological heterogeneity of colorectal cancer (9), it is important to search for a *method for targeted biopsy sampling* (10, 11). Targeted biopsy sampling will significantly increase the effectiveness of subsequent morphological examination and will allow a comprehensive study of morphological and molecular heterogeneity of colorectal cancer (12). Colorectal cancer *morphological subtype* and *degree of difference (grade)* between the cancerous tissue and the initial normal tissue established at histological examination affect further planning of personal therapy (13).

According to literature data, 40-90% of all colorectal cancer cases belong to the morphological subtype of colorectal adenocarcinoma not otherwise specified (CRAC) (7, 14) while the rest 10-60% belong to other morphological subtypes and are often not diagnosed in clinical practice. These low rates of diagnostics are related to the tumor’s pronounced morphological heterogeneity, insufficient knowledge about the characteristic features of rare colorectal cancer morphological subtypes and the lack of generally accepted recommendations for treatment of each colorectal cancer morphological subtype (15). At the same time, research studies have shown that correct identification of morphological subtypes of colorectal cancer may be crucial for selecting proper treatment (7). For example, mucinous adenocarcinoma is associated with frequent loco-regional lymph node involvement (7). The presence of cribriform CRAC pattern is often related to a high frequency of venous invasion, organ metastasis and cancer cells mutation (16–18). Similarly, mistakes and failures occur at the stage of postoperative material collection and analysis, which are often made regardless of colorectal cancer morphological subtype and without paying attention to rare subtypes. However, such mistakes could be avoided if the tumor morphological subtype is closely identified after the collection of morphological material (7).

According to the World Health Organization classification of tumors of the digestive system, the grade of CRAC differentiation varies from highly or moderately differentiated (low-grade, Grade I-II) to poorly differentiated (high-grade, Grade III) or even undifferentiated (Grade IV) (19). It should be noted that the grade of CRAC differentiation has been indicated in numerous studies as a decisive factor for prognosis and choice of treatment tactics (20–22). According to statistics, patients with poorly differentiated CRAC are characterized by a higher risk of recurrence after tumor resection, which significantly deteriorates the prognosis (23). It is known that preoperative radiation or chemoradiation therapy can significantly improve the prognosis and survival of patients with poorly differentiated CRAC and high risk of recurrence (24). However, it is also known that the use of neoadjuvant therapy may lead to serious side effects – from the decrease in anorectal function (25) to the appearance of secondary cancers in organs near the primary tumor (26). Therefore, the ability to distinguish low-grade from high-grade CRAC before the initiation of treatment, and identify patients with a high risk of recurrence, can increase the quality of patient’s life and post-treatment lifespan by maximizing the treatment benefit and avoiding undesired side effects.

For identifying the exact location of tumor node and malignant morphology, we propose using the new method of Compression Optical Coherence Elastography (C-OCE). Based on successful experience of applying Multimodal Optical Coherence Tomography (OCT) in studying morphology of other oncological diseases [mainly, breast cancer (27, 28) and gliomas (29)], we may assume the effectiveness of C-OCE for colon tumor localization detection and consequent identification of colorectal cancer differentiation grades and morphological subtypes. At the same time, despite its limitation of the scanning depth (up to 2 mm), we expect that the C-OCE technology can be efficient, because usually colorectal cancer proliferates and progresses from mucosal cells (on inner surface lumen of colon wall) (2), which well corresponds to the C-OCE visualization depth during transrectal examination. Endoscopic access to colon wall may help the new C-OCE technology to assess colorectal cancer morphology. Taking into account past experience (30, 31), C-OCE has prospects of becoming a clinical tool for endoscopic morphological diagnosis of colorectal cancer heterogeneity before targeted biopsy. Considering the above arguments, the *aim* of this study was to determine the ability of C-OCE to detect colorectal cancer and to distinguish grade of differentiation and morphological subtypes of colorectal cancer. To this end, we examined postoperative colon tissue samples using C-OCE to identify characteristic and distinctive features of: (i) *normal* and *benign tumorous colon tissue*, (ii) *malignant tumorous colon tissue*, (iii) *low-grade* and *high-grade CRAC* and *specific morphological features of colorectal cancer tissue*.

## 2 Material and methods

### 2.1 Colon sample characteristics

The study was carried out in accordance with international and ethical standards set out in the World Medical Association Declaration of Helsinki “Ethical principles for medical research involving human subjects” (32). The present study was approved by the Institutional Review Board of the Privolzhsky Research Medical University (REB#3 of February 21, 2020). Informed consent was obtained from all participants. Human colon samples were collected at the Volga District Medical Centre of Federal Medical Biological Agency of Russia from patients during surgery.

The study included 19 patients who had been diagnosed with colorectal cancer based on the results of a histological examination of colon wall biopsies taken during total colonoscopy. All the patients underwent physical examination, laboratory diagnostics (complete blood count, including carcinoembryonic antigen, urinalysis) and instrumental diagnostics (computed tomography of the lungs, abdominal cavity and pelvis and MRI of the abdominal cavity to identify the spread of cancer to the liver) according to RUSSCO Clinical Practice Guidelines (33), corresponding to generally accepted guidelines (3). According to the results of MRI with intravenous contrast, foci of colorectal metastases to the liver were identified in 3 patients. These patients underwent extended surgery where elective liver resections were also performed for colorectal liver metastases. All the patients enrolled in the study had not been pretreated with radiotherapy or chemotherapy.

**Figure 1:**
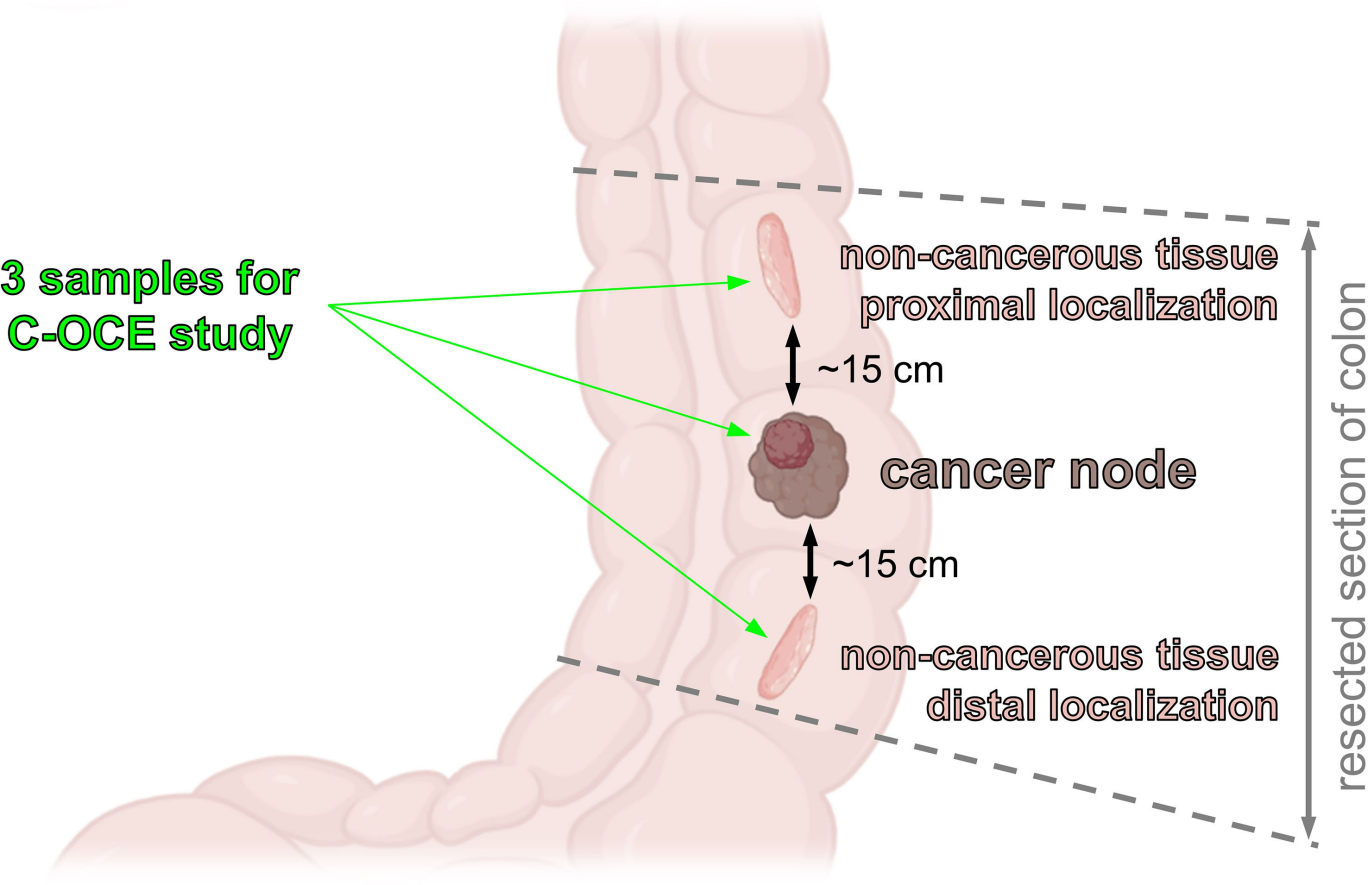
Colon samples localization for C-OCE study.

From each patient, 3 regions of interest (ROI) were measured with C-OCE: by one sample of cancerous colon tissue (n=19) taken from the centre of tumor area and by two samples of non-cancerous tissue (n=38) taken from areas located at a distance of ∼10-20 cm distal and proximal from the tumor node (**Figure 1**). The total number of studied samples was 57. The size of samples varied from 15 mm to 27 mm. The samples were delivered to the laboratory in 10% BSA on ice within 30 minutes after resection and were studied within 30-70 minutes after resection.

According to the established morphological subtypes of colorectal cancer (34), all the cases were divided into 3 groups: CRAC low-grade [well differentiated and moderately, Grade I-II] (n=11), CRAC high-grade [poorly differentiated, Grade III] (n=5) and mucinous adenocarcinoma (MAC; n=3). Among CRAC subtype in 4 samples a pathologist noted the presence of a cribriform pattern – tumor glandular structures of a lattice shape with a wide lumen, in which areas of comedo necrosis were identified (35). Among the samples of non-cancerous colon tissue, in 5 cases areas of epithelial hyperplasia of mucosa were detected. These samples were attributed to benign colorectal adenoma. The remaining 33 samples were presented with normal morphology (**Table 1**).

**Table 1:** Clinical and pathological characteristics for the examined cohort.

Patient characteristics (n=19)								
Gender	Men (n=14)			Women (n=5)			
Age	62 ± 13 [37; 82]							
Cancer location	splenic flexure (n=1)		descending colon (n=6)		sigmoid colon (n=9)		rectosigmoid junction (n=3)	
Tissue samples characteristics (N=57)								
Tissue type	Non-cancerous tissue (N=38)			Cancerous tissue (N=19)				
Morphological group	Normal colon (N=33)	Colorectal adenoma (N=5)		CRAC				MAC (N=3)
				Low-grade (N=11)		High-grade (N=5)		

### 2.2 Compression optical coherence elastography

#### 2.2.1 Multimodal OCT system

**Figure 2:**
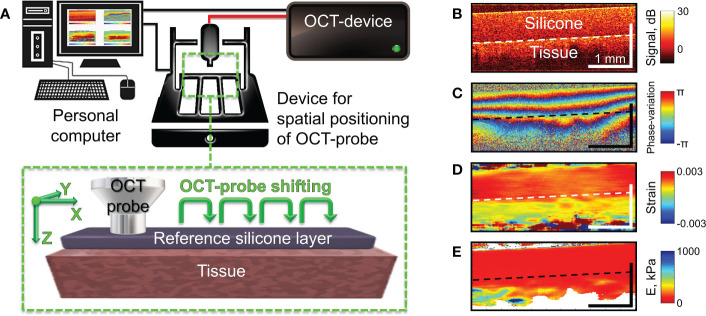
Schematic of C-OCE examination and the main stages of C-OCE processing. **(A)** – OCT scanner with the positioning device to enable acquisition of several maps of the Young’s modulus for subsequent stitching in the lateral direction. The reference layer of translucent silicone placed onto the tissue is used for stiffness quantification. **(B)** – a typical structural OCT scan for the sandwich structure “silicone-tissue”. **(C)** – a typical map of interframe phase variation obtained during compression of the tissue through the overlying silicone layer. **(D)** – spatial distribution of interframe strains reconstructed from the phase-variation map shown in panel **(C, E)** – resultant map of tangent Young’s modulus reconstructed from spatially-resolved stress-strain dependences obtained using a series of several tens OCT scans acquired during compression of the tissue for a fixed lateral position of the OCT-probe. The Young’s modulus for every lateral coordinate was estimated for the applied stress of 4 kPa which was controlled by the value of strain in the overlaying reference silicone as described in (36).

The studies were performed using a spectral-domain multimodal optical coherence tomograph (**Figure 2A**) developed at the Institute of Applied Physics of the Russian Academy of Sciences (Nizhny Novgorod, Russia) with a central wavelength of 1310 nm, power output of 15 mW, lateral resolution of 25 µm, axial resolution of 15 µm, scanning depth of up to 1.5 mm and scanning speed 20.000 A-scans per second (37). After making a recording (26 seconds for structural OCT), we obtained a 3D array of speckles of different intensities with 4.0×4.0×2.0 mm dimension, where the central B-scan was analyzed. For targeted co-positioning of the OCT B-scans and the subsequent histological slides, a 3D positioning system – Purelogic R&D PLRA4 (Russia) was used. It performs 3D positioning of the OCT-probe with the accuracy of 10 μm. The use of this setup enabled both high-precision lateral positioning, as well vertical movement of the probe required for performing controlled compression of the studied samples during C-OCE examinations (**Figure 2B**). For increasing the size of the examined region, a series of aligned B-scans can be obtained as shown in **Figure 2A** and then stitched, yielding the resultant lateral size of OCT images ~several tens’ millimeters.

#### 2.2.2 C-OCE imaging

Since reliable OCT-based correlational mapping of local strains requires super-broadband optical sources due to strain-induced decorrelation of compared OCT images (38), we utilized a phase-resolved variant of C-OCE (39). In the used C-OCE variant, the interframe strains were calculated by estimating axial gradients of the interframe phase variation using the “vector method” (40, 41). The method is termed “vector” because it treats complex-valued signal amplitudes as vectors in the complex plane and the signal phase is explicitly singled out at the very last processing stage. This method is very robust with respect to various measurement noises and strain-induced decorrelation and does not require phase unwrapping along the B-scan depth allowing to measure interframe strains up to ~1%. **Figures 2B–D** demonstrate a typical structural OCT scan, an example of interframe phase-variation and the corresponding interframe strain found by the vector method. For finding even larger strain values, cumulative strains for a series of elastographic frames can be found (42). In the course of strain estimation, the numerical differentiation of the depth-dependence of interframe phase variations is performed with a certain averaging to obtain stabler results and suppress the measurement errors. In practice, the typical size of the averaging window is 80-100 μm both laterally and axially. The resultant spatial resolution of strains maps (similar to the example shown in **Figure 2D**) is ~1/2 of the processing window size, i.e., 40-50 μm.

Next, to enable quantification of the Young’s modulus (stiffness), a pre-calibrated reference silicone layer is placed on the surface of the biological tissue (43–45) as shown in **Figure 2A**. It was carefully verified that silicone is a highly linear material, so that even rather large cumulative strains (~tens of per cent) remain linearly proportional to the stress compressing the silicone layer (42, 44). The silicone-tissue sandwich is compressed by the output window of the OCT probe and a series of OCT scans (typically, about a hundred) are acquired during the compression. Then by processing the acquired record, gradually increasing cumulative strains are estimated. By plotting the strain in the reference silicone (which is linearly proportional to stress) against the tissue strain, one obtains spatially-resolved stress-strain dependences for the studied tissue. For the majority of real tissues, their stress-strain dependences are pronouncedly nonlinear (see (44, 46, 47), so that usually the slope of the stress-strain curves (i.e., the tangent Young’s modulus) strongly changes its value (several times and more) when the strain varies rather moderately (within several per cent) or when the applied stress varies within several kPa. Therefore, to enable meaningful comparison of stiffnes in various measurements, the tangent Young’s modulus should be estimated for the same loading conditions. Since strain in real heterogeneous tissues usually vary in space quite pronouncedly (see **Figure 2D**), it is more practical to utilize the same applied stress which can be fairly conveniently estimated *via* the strain in the overlaying reference silicone. In such way the spatially-resolved estimation of the tangent Young’s modulus can be made by comparing strains of the precalibrated silicone and the examined tissue for the same pre-chosen stress applied to the tissue over the entire OCT frame in each OCE examonation. To this end, a special procedure based on local stress estimation was developed [see details in (36)] using which we can form elastographic stiffness maps corresponding to a preselected standardized pressure value. For the presented below quantitative estimates of Young’s modulus, we used the pressure range from 3 kPa to 5 kPa centered at 4 kPa. The reconstructed distibution of the Young’s modulus corresponding to panels in **Figure 2B–D** is shown in **Figure 2E**, in which the “standardized” applied stress over the scan is equal to 4 kPa. The choice of this stress value is based on the vast previous experience of performing OCE examination of various tissue samples. Rougly such stress corresponds to strains of the order of several per cent (certainly somewhat different for different tissues). Such strains are measure by summation of several tens of incremental interframe strains, which helps to significantly improve signal-to noise ratio in the resultant strain maps (42). Furthemore, often morphological components of the studied heterogeneous tissue demonstrate higher contrast in stiffness for such intermediate stress of 3-5 kPa rather than for significantly smaller or greater stresses.

### 2.3 Histological study

During resection and subsequent examination of samples by C-OCE and histology, spatial positioning of the sample was important – OCT probe was placed to the mucosa, so the study “simulated” endoscopic examination of colon lumen. Immediately after the C-OCE study, postoperative tumor samples were subjected to histological examination. Tumor samples were fixed in 10% formalin for 48 hours. Then several (~4) serial sections with thickness of 7 μm were made along the direction coinciding with the C-OCE-scan position. For such co-location, the positions of the C-OCE scans were marked on the surface of the studied samples using histological ink (Histo-Line, red). All histological sections were stained according to the standard technique with hematoxylin and eosin (H&E), which made it possible to assess the tissue microstructure.

### 2.4 Processing of C-OCE and histological data

To determine stiffness value range for each morphological structure, representative areas in size 110x110 µm were selected in histological images, located within the histological structure region, but not near the border. Then the histological images were compared with the corresponding C-OCE images which were square areas of 7×7 = 49 speckles. Larger areas were not examined due to the frequent proximity to the border of histological structures. For all histological structures, at least 30 square areas (~1500 speckles in total) were analysed, which was enough to determine the total spectrum of stiffness values (28). Automatic segmentation algorithms described and applied earlier (28) were used to segment the obtained C-OCE images into areas of cancer cells and other tissue components. The threshold value for cancer cells segmentation was set at the highest correlation of areas on histology and C-OCE images.

**Figure 3:**
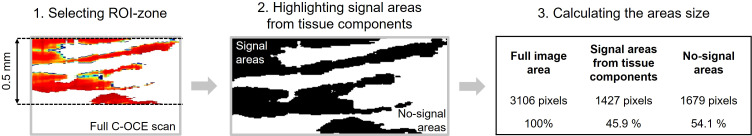
Isolation and quantification of non-signal regions in C-OCE images for differentiating MAC and CRAC with cribriform pattern.

The C-OCE images of MAC and CRAC with cribriform pattern were characterized by areas of signal loss due to the lack of scattering elements of tissue components; moreover, to establish MAC, non-signal areas by extracellular mucin must be at least 50% of the total tissue area (34). Therefore, an approach was developed to determine the non-signal areas sizes of C-OCE images for differentiating MAC and CRAC with cribriform pattern samples. The signal most often did not penetrate to the depth of more than 500 μm, based on which it was decided to count non-signal areas of ROI no deeper than this value (**Figure 3**). Areas with stiffness values ≥ 1 kPa, were highlighted in black by ImageJ 1.8.0 software (NIH, USA). Non-signal areas retained white coloration. Next, the ratio of non-signal areas and areas with stiffness values ≥ 1 kPa in ROI was calculated.

The statistics of average stiffness values of some histological structures included values of several morphological subtypes: for tumor stromal fibres – samples of CRAC and MAC, for glandular cancer – samples of low-grade CRAC including cribriform pattern. This is due both to absence of significant differences in morphology of such structures among different subtypes, and to very similar defined (in this study) stiffness value ranges of such structures among different subtypes.

### 2.5 Statistical analysis

Statistical analysis was performed in the Statistica 10.0 (Tusla, OK, USA) and the GraphPad Prism 8.0 (San Diego, CA, USA) using the one-tailed Student’s t-test. All results of C-OCE metrics are expressed as ‘Mean ± Standard Deviations [Max.; Min. Values]’. Pearson’s correlation coefficient was used for comparison between areas of cancer cells determined by morphological segmentation of histology images and from the automatic segmentation of C-OCE images. P-values less than 0.05 were considered statistically significant. Besides, the Bland–Altman analysis (48) was performed to highlight similarities and differences between measurements by C-OCE and histology (reference standard). Using this analysis allows one to demonstrate if C-OCE method overestimates high values or underestimates low values. Also, the results are represented graphically by the distribution of the scatter plot with indicating value of bias (mean of systematic error) and 95% limits of agreement.

## 3 Results

### 3.1 Study of normal colon tissue and benign adenoma

**Figure 4:**
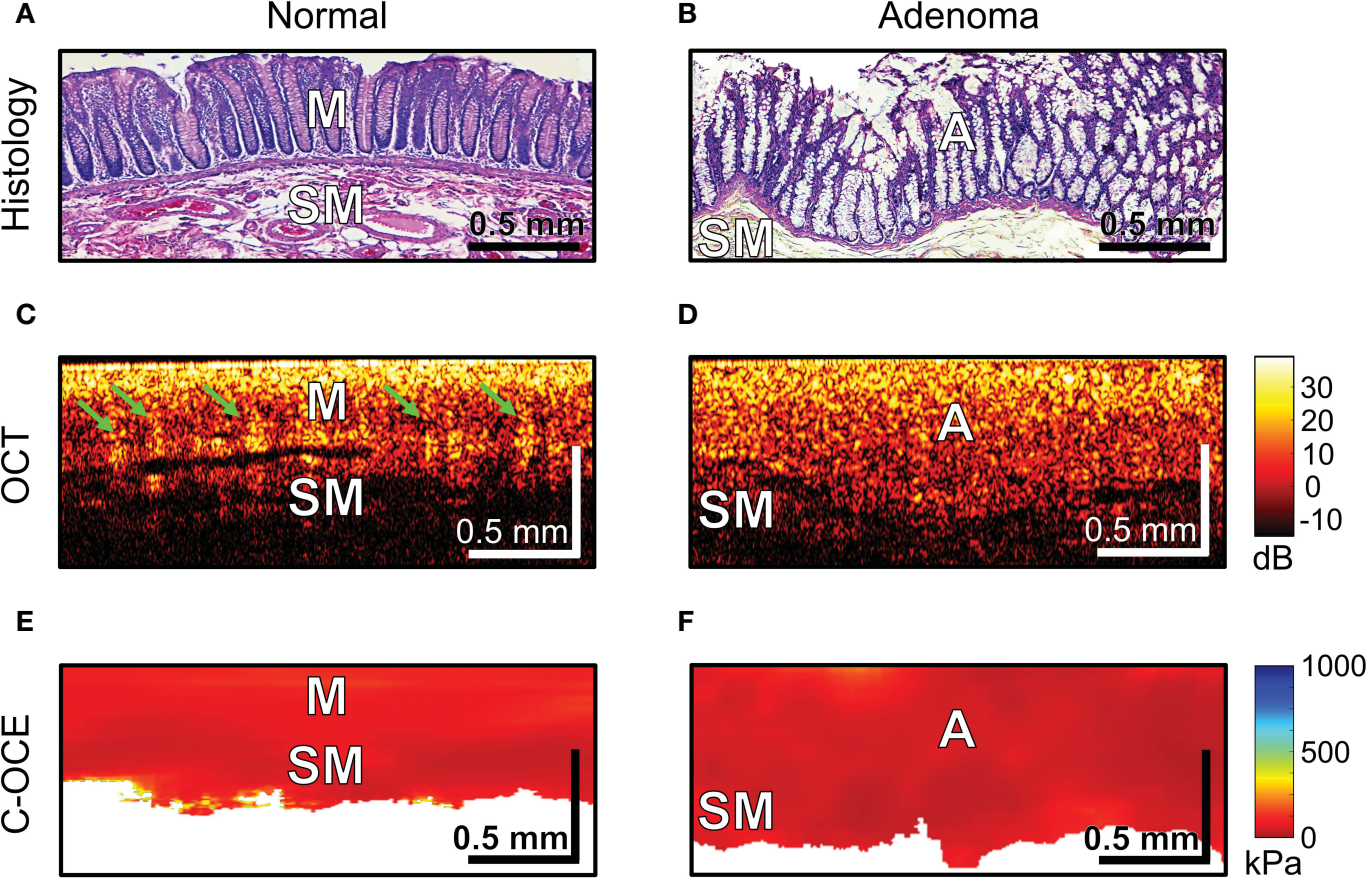
Examples of morphological examination of normal colon **(A, C, E)** and colorectal adenoma **(B, D, F)** tissues by C-OCE: low stiffness values of mucosa [M] (58 ± 28 kPa) and submucosa [SM] (43 ± 17 kPa) did not statistically significantly differ from stiffness values of benign adenoma [A] (46 ± 22 kPa). Corresponding tissue areas in histological **(A, B)**, OCT **(C, D)** and C-OСE **(E, F)** images. Bar size is shown in the images.

The first step involved the analysis of normal colon tissue and benign colorectal adenoma. The capabilities of structural OCT and C-OCE visualization of colon tissue and limitations of the scanning depth were determined. Comparison with histological sections of *normal colon* wall (**Figure 4A**) showed that in structural OCT images mucosa and submucosa are well distinguished: in mucosa regular vertical features corresponding to crypts are present which form a «teeth» pattern of this layer (**Figure 4C**, green arrows); submucosa has a porous heterogeneous structure (**Figure 4C**) due to presence of blood and lymphatic vessels, and nerve fibres, surrounded by connective tissue (**Figure 4A**). Attenuation of OCT-signal is observed at the level of submucosa so that the underlying muscularis propria is not visualized. C-OCE shows that mucosa and submucosa have a similar levels of stiffness (**Figure 4E**). Mucosa is homogeneous in stiffness distribution and has an average stiffness value of 58 ± 28 [23; 104] kPa. Submucosa is also homogeneous in stiffness distribution and has a lower average stiffness value of 43 ± 17 [21; 77] kPa. In some cases, we did not receive a useful signal in C-OCE images by colon submucosa. This may be due to difficulty in tracking small morphological structures (blood and lymphatic vessels, nerve fibers, connective tissue) of submucosa during their compression (morphological structures shift and change their localization – escape from the detection window).

*Colorectal adenoma* which is histologically characterized by mucosal hyperplasia and chaotic growth of colon crypts (**Figures 4B**), in structural OCT images showed the absence of a “teeth” pattern in mucosa (**Figures 4D**) and noticeable increase in mucosa thickness both in structural OCT (**Figures 4D**) and C-OCE (**Figures 4F**) images. A general increase in scanning depth is also observed. Colorectal adenoma is homogeneous in stiffness distribution and has an average stiffness value of 46 ± 22 [19; 84] kPa (**Figure 4F**), which is lower than stiffness values of normal mucosa. However, the stiffness of colorectal adenoma tissue did not statistically significantly change (p >0.05) compared to stiffness of normal mucosa. Thus, based on comparison of characteristic stiffness ranges only it is not possible to differentiate normal colon tissue (mucosa and submucosa) from benign colorectal adenoma for which fairly similar low stiffness values are found. Nevertheless, it is possible to distinguish benign colorectal adenoma from normal colon tissue using structural OCT images in which the adenoma looks fairly homogeneous, whereas the normal tissue is more stratified (has two layers) and exhibits very peculiar “teeth” structures in mucosa.

### 3.2 Identification of colorectal cancer

**Figure 5:**
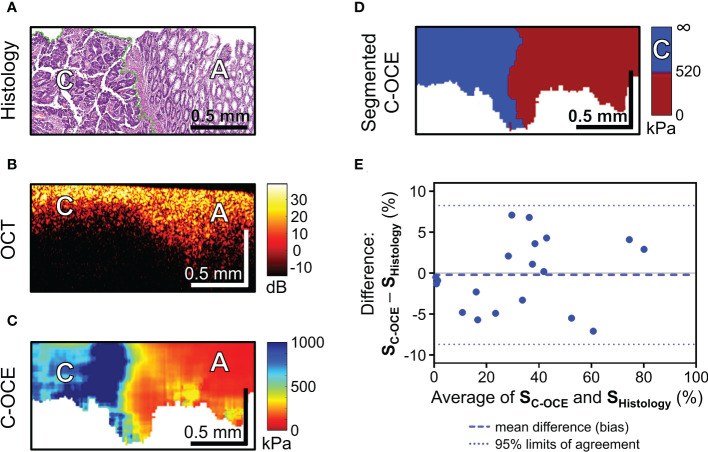
C-OCE identification of colorectal cancer border with non-tumorous tissue and correlation between C-OCE and histological data. Corresponding tissue areas in histological **(A)**, OCT **(B)** and C-OСE **(C)** images. **(D)** – segmented C-OCE image derived from stiffness C-OCE image **(C)** using the color palette and the characteristic stiffness of colorectal cancer cells >520 kPa; designations: [C] – cancer cells, [A] – adenoma bar size is shown in the images. **(E)** – quantitative comparison of histological and C-OCE-based segmentation results presented in the form of a Bland Altman diagram. The diagram reflects the dependence of the established averages between the two methods of knowledge and the difference between the established values of the areas of colorectal cancer (SC-OCE – S histology, %); symbols: blue dotted line – bias (mean of systematic error), blue dotted lines – upper and lower 95% limits agreement. Additionally, a comparison between the areas of cancer cells identified by morphometric analysis by C-OCE and histology, shows a strong and direct correlation (Pearson correlation coefficient r =0.98; p <0.05).

During the next stage of the analysis, we examined *colorectal cancer* tissue samples. In our study of malignant tumorous colon tissue in structural OCT images, a slight decrease in depth of signal penetration (**Figures 5A, B**) and complete absence of “teeth” structures (mucosa crypts) were observed. However, in the C-OCE images sharply inhomogeneous stiffness distribution was observed, where areas of increased stiffness values (>500 kPa) were found (**Figure 5C**). This was not observed in any cases of non-malignant colon (**Figure 4**). Such qualitative differences between malignant tumor and benign tumor/normal colon tissue indicate the possibility of identifying the threshold stiffness value, and values above it would signify the presence of colorectal cancer cells at the C-OCE detection site.

After a targeted comparison areas with cancer cells in histological and corresponding C-OCE images, the range of occurring stiffness values for colorectal cancer cells 520-1418 kPa was established and, consequently, a threshold stiffness value of 520 kPa was chosen for subsequent segmentation of C-OCE images. The resulting segmented C-OCE images (**Figure 5D**) were highly consistent with the histological images. In addition to the very close results of the two methods of cancer cell detection, a high correlation was demonstrated between the area of cancer cells highlighted by the pathologist in the routine way on the histological section and the area segmented in the C-OCE image according to the corresponding stiffness value range. Pearson correlation coefficient between the areas occupied by colorectal cancer cells in the histological images and the segmented C-OCE image is very high, r =0.98 (p <0.05). In addition, according to the results of the Bland-Altman statistical analysis (**Figure 5E**), the identified 95% limits agreement are quite narrow (-8.27% lower limit, and 8.70% upper limit) and the bias (-0.22 ± 4.33%) is negligible, which allows us to conclude that the two methods (C-OCE and Histology) are essentially equivalent in morphometry of colorectal cancer cell areas. It should also be noted that we identified no dependence of overestimation or underestimation of the values of the determined areas of cancer cells from the value of those in the studied samples by the C-OCE method. Also, overestimation or underestimation of values was not found to be associated with the morphological structure and subtypes of the studied colorectal cancer.

To sum up, according to the results of colorectal cancer tissue sample study, it is important to note a significant quantitative increase in stiffness values when detecting malignant tumor tissue in C-OCE images. When comparing histological images and C-OCE images, colorectal cancer cells (for any of the studied morphological subtypes) were determined in the range of stiffness values above 520 kPa. Segmentation of cancer cells in C-OCE images in this range showed a high correlation and correspondence of results with morphological segmentation of histological images – r =0.98 (p **<**0.05). Thus, the results of morphological segmentation of C-OCE images based on cancer cell stiffness are highly consistent with the results of histological examination, which confirms the possibility and the potential of using C-OCE to determine precise localization of cancer and subsequent targeted biopsy sampling. The next step of the study aims at identifying the characteristic of cancer morphology by C-OCE.

### 3.3 Distinguishing differentiation grade and morphological subtypes of colorectal cancer

**Figure 6:**
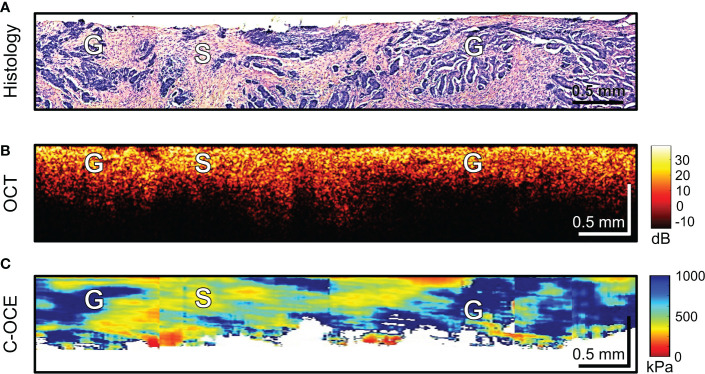
Example of morphological examination of low-grade colorectal adenocarcinoma (low-grade CRAC) tissues by C-OCE: stiffness values of tumorous stroma [S] are lower (295 ± 120 kPa) than in gland-like structures [G] (724 ± 86 kPa). Heterogeneity in the spatial distribution of this two tissue types is observed. Corresponding tissue areas in histological **(A)**, OCT **(B)** and C-OСE **(C)** images. Bar size is shown in the images.

**Figure 7:**
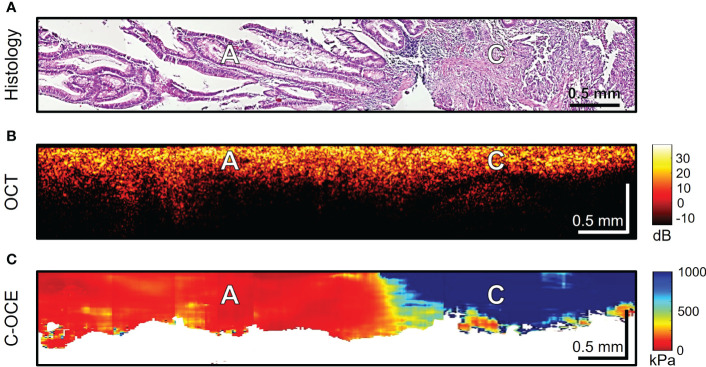
Example of morphological examination of high-grade colorectal adenocarcinoma (high-grade CRAC) tissues by C-OCE: non-glandular cancer tissue [C] has very high stiffness (954 ± 156 kPa) in comparison with adenoma [A] (46 ± 22 kPa). Corresponding tissue areas in histological **(A)**, OCT **(B)** and C-OСE **(C)** images. Bar size is shown in the images.

The last stage of the study involved differentiating between low-grade and high-grade CRAC, feature identification of CRAC cribriform pattern and MAC mucin fields in the obtained C-OCE images. First, tissue samples of colorectal cancer diagnosed as morphological subtype CRAC were examined. Histological examination of *low-grade CRAC* tissue samples revealed multiple gland-like structures occupying more than half of histological section area and located among the stromal fibers (**Figure 6A**). Structural OCT images showed no significant qualitative differences between areas of tumor structures and stroma (**Figure 6B**). In turn, in C-OCE images gland-like structures (glandular cancer) were characterized by high stiffness values – 724 ± 86 [520; 944] kPa, while stroma had lower values – 295 ± 120 [92; 515] kPa (**Figure 6C**). Statistics of mean stiffness values for stroma and gland-like structures include obtained values for other colorectal cancer morphological subtypes (see explanations in Methods/Processing of C-OCE and histological data). Histological examination of *high-grade CRAC* tissue samples revealed foci of dense conglomerates of cancer cells densely infiltrated with lymphohistiocytic cells (**Figure 7A**). We did not find gland-like structures in such samples. As it was mentioned above, structural OCT images did not have pronounced qualitative differences between cancer cells and colon mucosa (**Figure 7B**); only a slight decrease of signal penetration depth was noted. During the examination of these tissue samples by C-OCE, qualitatively homogeneous areas of very high stiffness (954 ± 156 [703; 1418] kPa) values in foci of cancer cells (non-glandular cancer) projection were often identified (**Figure 7C**).

**Figure 8:**
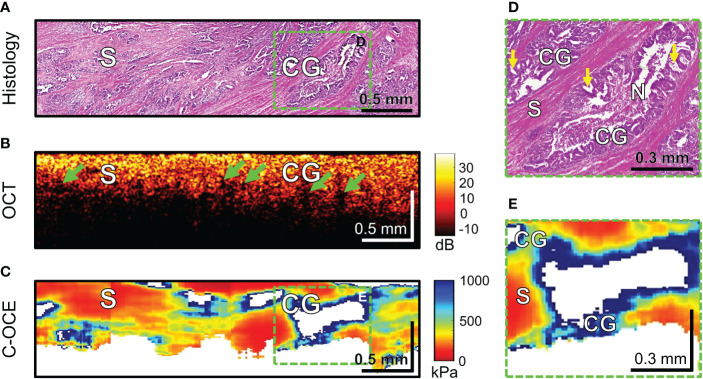
Example of morphological examination of colorectal adenocarcinoma with cribriform pattern tissues by C-OCE: cribriform gland-like structures [CG] characterized by high stiffness values (724 ± 86 kPa) and located among stroma [S] having lower stiffness (295 ± 120 kPa). Comedo necrosis [N] was not detected by C-OCE due to its free presence in the gland-like structures’ lumen. Corresponding tissue areas in histological **(A)**, OCT **(B)** and C-OСE **(C)** images. Green arrows in panel B indicate faintly visible areas of signal loss associated with a large lumen of cribriform gland-like structures. Enlarged areas of cribriform gland-like structure on histological **(D)** and C-OСE **(E)** images demonstrate nodular nests with ‘punched out’ spaces (yellow arrows) as well as intraluminal bridging. Bar size is shown in the images.

Further, the morphological patterns of colorectal cancer tissue, which were characterized by a specific structure in the C-OCE images, were examined. Histological examination of four CRAC samples identified *cribriform pattern* areas tissue (**Figure 8A**) – extensive cribriform gland-like structures (cribriform architecture on **Figure 8D** marked with yellow arrows) with a wide lumen where central comedo necrosis is located. OCT images are not visually characterized by cribriform gland-like structures. Such structures with large lumen are characterized by loss of OCT-signal inside images (marked with green arrows in **Figure 8B**). This may be due to incomplete filling by necrosis of the free space of gland-like structure’s lumen. The areas of C-OCE images corresponding to cribriform gland-like structures (**Figures 8C–E**) were characterized by high stiffness values (in range from 531 to 928 kPa) that did not go beyond values of low-grade CRAC gland-like structures. The statistics of obtained stiffness values of cribriform gland-like structures was included in the group of glandular cancer by low-grade CRAC (as described in Methods/Processing of C-OCE and histological data) due to the high similarity of morphological structure of glandular-like structures tissue and detection of similar ranges of stiffness values. Comedo necrosis was not detected by C-OCE due to its free presence in the gland-like structures’ lumen (**Figure 8D**). Signal loss is observed in C-OCE images in projection of gland-like structures lumen (**Figure 8E**), which confirms the above. The gland-like structures were located among stromal fibers, the areas of which in the C-OCE images were characterized by lower stiffness values (**Figure 8C**) which were in the same range as given above for CRAC samples.

**Figure 9:**
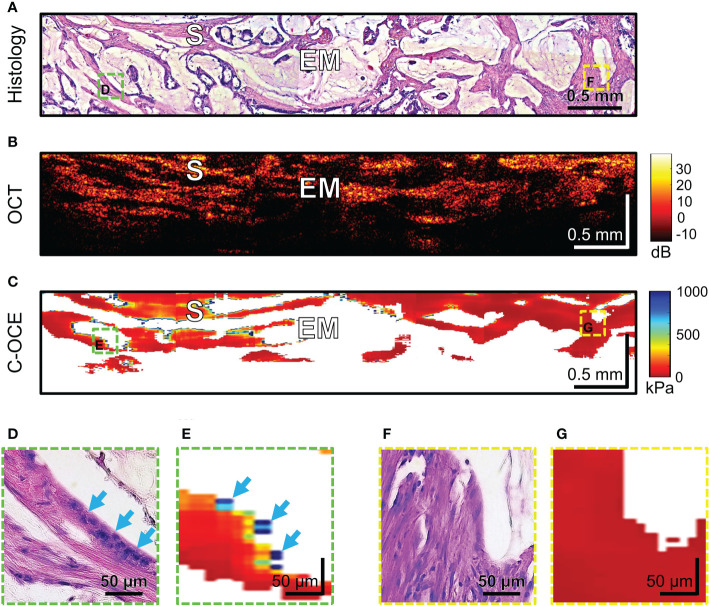
Example of morphological examination of mucinous adenocarcinoma (MAC) tissues by C-OCE: abundant accumulations of extracellular mucin [EM] with absence of measured stiffness (non-signal areas) are clearly separated from tumorous stroma [S] having stiffness values around 295 ± 120 kPa. Corresponding tissue areas in histological **(A)**, OCT **(B)** and C-OСE **(C)** images. Enlarged areas **(D-G)** of tumor tissue in histological **(D, F)** and C-OСE **(E, G)** images demonstrate presence **(D, E)** or absence **(F, G)** of small clusters of cancer cells, including signet ring cells (blue arrows) attached to stromal fibers. Bar size is shown in the images.

Histological examination of *MAC* samples demonstrates abundant extracellular mucin (more than 50% of tumor area) associated with ribbons or tubular structures of neoplastic stroma (**Figure 9A**). Clusters of cancer cells, including signet ring cells, not infrequently may be found attached to the adjacent stromal walls (**Figure 9D**, blue arrows), but often cells floating within the mucin and stromal wall can be free of tumors’ infiltration (**Figure 9F**). OCT images cannot qualitatively display small clusters of cancer cells, however, mucin fields are clearly visualized as areas of useful OCT-signal loss, which are limited by stromal walls (**Figure 9B**). During C-OCE study, however, images identified mucin fields (loss of signal by C-OCE caused by low back scattering power of components mucin) and stromal walls (as by C-OCE – middle and low stiffness values, which did not differ from previously studied stroma in other colorectal cancer samples) (**Figure 9C**). Some discrepancy between histological and C-OCE images is associated with a significant change in the topology of stromal walls in mucin fields during compression of studied tissue for C-OCE-detection. In several cases, clusters of cancer cells on stromal walls were observed (**Figure 9D**), which were represented in the C-OCE images by one or two adjacent speckles of high stiffness values (**Figure 9E**). The high values of such speckles were determined in stiffness range of non-glandular tissue (similar to cases of high-grade CRAC). However, more often stromal walls free from cancer cells invasion with low stiffness values in C-OCE images were observed (**Figures 9F, G**). The most striking difference of MAC from others morphological subtypes is the loss of signal in more than 50% of C-OCE image area (**Figure 9C**).

In summary, in C-OCE images, the heterogeneous distribution of speckles with stiffness values 520-950 kPa corresponds to low-grade CRAC, and the homogeneous distribution of speckles with stiffness values above ~950 kPa corresponds to high-grade CRAC. The main distinguishing feature of the considered colorectal cancer from other subtypes is the loss of signal from some structures of tumor tissue. In C-OCE images, the visualization of the CRAC cribriform pattern is similar to the low-grade CRAC with the exception of loss of signal in the center of gland-like structures in the CRAC with cribriform pattern. The main distinctive feature of the MAC subtype is the large-scale predominance of signal loss areas from weakly scattering mucin.

## 4 Discussion and conclusion

To our knowledge, this is the first *ex vivo* study to evaluate the diagnostic capability of C-OCE for human colon tissue. It is worth noting that the majority of earlier OCT studies demonstrated the ability to distinguish normal colon tissue from polyps (49)/neoplasia (50)/and cancer (51–53) based only on structural data. The proposed solutions in most cases are aimed at optimizing the targeted tissue sampling for subsequent histological examination. In addition, it has already been shown that the use of an additional modality such as cross-polarization OCT for *en face* mapping allows successful identification of colon tissue neoplasia (54). The joint use of near-infrared fluorescence with OCT, for example, to visualize the structure and microvasculature of the colon demonstrated the possibility of identifying cases of early stages of colorectal cancer (55). Therefore, further development of OCT modalities and their joint application may make it possible to determine the most effective and objective quantitative parameters for the diagnosis of colorectal cancer, which have prospects for clinical application for the tasks of morphological examination of tissue within colonoscopy for targeted biopsy.

In our work, we once again demonstrated the characteristic structural features of normal and pathological tissues described earlier (49). In particular, in structural OCT images of non-neoplastic colon tissue two layers could be clearly distinguished: mucosa and submucosa. In colon mucosa, glandular crypts were moderately distinguishable and characterized by “teeth” architectonics. In colon submucosa, layered heterogeneous structures (blood and lymphatic vessels, nerve fibers, surrounded by connective tissue) with a gradual signal attenuation with depth were observed. Hyperplastic benign adenoma was characterized by mucosal overgrowth, which increased the thickness of the layer and almost complete disappearance of the signal from the submucosa in structural OCT images; “teeth” structures were indistinguishable. The structure of malignant tumorous colon tissue did not always visually differed from non-cancerous colon tissue in terms of signal depth penetration; however, qualitative difference was also found in the absence of “teeth” structure in OCT images of colorectal cancer. The characteristic qualitative features, such as the absence of serrated architectonics and the disappearance of layering, were inextricably linked with the pathological processes of the colon, as was previously shown in the OCT examination of the colon by our research group (31) and others (56). However, in order to optimize qualitative diagnostic parameters of the OCT method for detecting cancer in benign and normal colon tissue, quantitative analysis parameters by deep learning-based pattern recognition were developed and applied recently (57–59). In these cases, machine learning relied on the disappearance of “teeth” structures (57), loss of layering (58) in hyperplastic or neoplastic processes of the colon. In addition, it is also worth noting the high efficiency of combining OCT with machine learning in the differentiation of colorectal liver metastases from liver parenchyma *ex vivo*, which is very important in the intraoperative examination of resection margins during liver surgery (60). Subsequently, significant progress has been achieved in colonoscopy of colorectal cancer using OCT and machine learning, which was demonstrated in a recent publication (59), where high sensitivity and specificity (~93%) were achieved in the detection of colorectal cancer. However, as the authors noted, such studies did not assess the OCT ability to distinguish other colorectal pathologies, such as mucosal adenoma, which, based on the characteristics of OCT detection and adenoma morphology, are likely to be erroneously assigned to cancerous areas of the colon.

In this regard, as our study demonstrates, C-OCE has great potential in differentiating benign adenoma from colorectal cancer. When analyzing C-OCE images of malignant tumorous and benign adenoma/non-tumor colon tissues, sharp qualitative differences in stiffness values were found. Colon mucosa was characterized by low stiffness values – 58 ± 28 [23; 104] kPa; and colon submucosa also was within low range values of 43 ± 17 [21; 77] kPa. Hyperplastic proliferation of mucosa by colorectal adenoma was noted to have stiffness values slightly lower than unchanged mucosa – 46 ± 22 [19; 84] kPa, which confirms the benign character of adenoma tissue. However, the absence of a statistically significant difference in the values of elasticity does not allow quantitative differentiation of hyperplastic benign adenoma from non-tumor colon tissues. An increase in the depth of the colorectal adenoma image obtained by C-OCE was not always clearly seen qualitatively, which indicates the need to use structural OCT to differentiate colorectal adenoma from normal colon mucosa, as shown in (54, 57) and in our study. At the same time, here we are first to demonstrate the minimum detectable stiffness value for malignant tumor tissue which was identified to be ~500 kPa. Qualitatively, by C-OCE images colon mucosa was represented in red color tone, and malignant tumor tissue – in turquoise or blue color tones. After comparing histological and C-OCE images, the range of encountered stiffness values for colorectal cancer cells was determined [520-1418 kPa] and a threshold value of 520 kPa was suggested for performing the automatic morphological segmentation described earlier (27, 28). When applying the segmentation algorithm of C-OCE images for morphometric assessment of colorectal cancer cells areas, a high correlation dependence for C-OCE morphometry and histological images was established with Pearson correlation coefficient r =0.98 (p <0.05). In addition, when plotting the Bland Altman diagram, the bias (-0.22 ± 4.33%) is negligible with rather narrow 95% agreement limits (-8.27% lower limit, and 8.70% upper limit), which indicates that the two methods (C-OCE and Histology) are essentially equivalent in morphometry of colorectal cancer cells areas. It is worth noting that more than 4–5 4 mm fields containing 512 A-scans were sequentially analyzed for each sample. In total, about 100 C-OCE images of cancerous tissue and about 200 C-OCE images of non-cancerous tissue were analyzed. In this case, only in 19 cancerous samples areas of stiffness values above 520 kPa were found, and in the remaining samples such high values were not found. For this kind of identification of colorectal cancer, C-OCE demonstrated its high level of accuracy. Thus, it can be concluded that C-OCE can both qualitatively and quantitatively identify the topography of colorectal cancer cells in accordance with the excess of stiffness values above 520 kPa. On the condition of future integration of C-OCE study into endoscopy, this technology can significantly assist in differentiating malignant colorectal cancer from benign colon adenoma and to accurately identify localization of colorectal cancer for targeted biopsy in clinical practice.

In modern clinical practice, MRI (4) and endoscopic ultrasound (61) are being actively used to obtain information about the morphological features of colorectal cancer tissue. High-resolution MRI is an established effective method for colorectal cancer staging due to its ability to diagnose colon wall laminar structure and show the details of the relationship of the tumor with the meso-rectal fascia and the surrounding organs (62). Also, MRI, along with endoscopy, plays an important role in assessing the response of a tumor to neoadjuvant therapy with an expected clinical complete response and choosing watch-and-wait patient management, where it allows assessing the thickness of the node or monitoring the structure of the bowel wall in case of a possible recurrence (63). Endoscopic ultrasound has even higher resolution, for which, in addition to the above, the possibility of performing highly accurate in-depth assessments of tumor infiltration (64), including cases of early colorectal cancer (65), and for the evaluation of subepithelial lesions (66) has been demonstrated. It is worth noting the integration into real-time endoscopic ultrasound of the elastographic modality (67), which has shown its effectiveness precisely in the context of morphological differentiation of malignant lesions from benign ones with high sensitivity/specificity (~0.93%) (68). This improves the quality of staging of colorectal adenomas and early cancers compared with the use of endoscopic ultrasound alone and may provide more accurate selection of patients suitable for local resection (69). One of the latest studies of endoscopic ultrasound elastography is related to the diagnosis of the depth of invasion of colorectal cancer (70), however, the resolution of ultrasound is insufficient to determine the morphological features of the structure of the tumor node at the level of individual morphological patterns, which is also not presented for any of the technologies discussed above.

The C-OCE method proposed in this paper, with the resolution of about 40-50 µm, made it possible to determine the qualitative differences of some morphological subtypes and tumor patterns: low-grade and high-grade CRAC (including CRAC with cribriform pattern), and MAC. In structural OCT images, low-grade and high-grade CRAC tissue samples did not differ qualitatively. Structural OCT images of CRAC with cribriform pattern and MAC tissue samples were distinguished by areas of signal loss inside tissue. Moreover, in the C-OCE images of CRAC with a cribriform pattern, these areas were hardly noticeable (detection due to wide lumen of cribriform gland-like structures), and for MAC tissue in C-OCE images areas of signal loss occupied more than 50% of image (detection due to large fields of extracellular mucin). When analyzing C-OCE images, some qualitative and quantitative features were identified for each morphological subtype and differentiation grade of colorectal cancer (**Table 2**). C-OCE areas of glandular and non-glandular cancer were characterized by different stiffness values – 724 ± 86 [520; 944] kPa and 954 ± 156 [703; 1418] kPa. Higher stiffness values of non-glandular cancer may be associated with denser localization of cells, a less pronounced intercellular connective tissue framework, or having a larger intercellular space within glandular cancer. Based on this, we can conclude that quantitative criterion for non-glandular cancer is the detection of stiffness values above 950 kPa. Thus, areas of very high stiffness (>950 kPa) in non-glandular cancer are found in high-grade CRAC and MAC samples. The low-grade CRAC C-OCE images were characterized by heterogeneous distribution of areas with increased stiffness (520-950 kPa) and middle stiffness (295 ± 120 [92; 515] kPa). These areas correspond to gland-like structure and stromal fibers, respectively, in histological images. Stroma can also be found in C-OCE images of high-grade CRAC and MAC, but along with middle stiffness stroma these images must show stiffness values higher than 950 kPa. Specific features of CRAC with cribriform pattern and MAC are the areas with absence of signal among areas of positive tissue signal. Moreover, in CRAC with cribriform pattern, such structures are located within areas of high stiffness values (from cribriform gland-like structures) and occupy much less than 50% of C-OCE image area. In MAC, such areas with absence of signal are extensive (more than 50% of C-OCE images area) and numerous. In future, we plan to study more surgical material in order to determine the precise elastic properties for differentiation of different grades and morphological subtypes of colorectal cancer, as well as to determine the diagnostic criteria for the C-OCE method in the detection of colorectal cancer. The identification of diagnostic criteria will allow comparison of the performance of C-OCE with other existing imaging modalities for detecting colorectal cancer, such as conventional optical coherence tomography, MRI, or endoscopic ultrasound. Besides, it could allow to create an algorithm for automated segmentation of C-OCE images for a simpler visual recognition of colorectal cancer morphological subtypes. Moreover, the development of an original endoscopic C-OCE-probe looks promising in the future and will make it possible to confirm/refute demonstrated effectiveness of this technology in clinical practice.

**Table 2:** A summary table of C-OCE characteristic features of normal colon, colorectal adenoma and each colorectal cancer morphological subtype.

Special C-OCE characteristics	Normal colon	Colorectal adenoma	Colorectal cancer			
			CRAC			MAC
			Low-grade	High-grade	Cribriform pattern	
Stiffness values 520-950 kPa	–	–	+	+	+	+
Stiffness values > 950 kPa	–	–	–	+	–	+
Areas of increased stiffness (gland-like structures)	–	–	+	–	+	–
Loss of signal (less than 50% of the image)	–	–	–	–	+	–
Loss of signal (more than 50% of the image)	–	–	–	–	–	+

It is worth emphasizing again that in this study we established the characteristic stiffness ranges using freshly excised samples of colon and then compared the results of segmentation of OCE images with conventional histological images segmented by an experienced pathologist. It cannot be excluded that *in vivo* the tissue may exhibit different elastic properties in comparison with even freshly excised samples. In future, we plan to experimentally verify this assumption by performing C-OCE measurements during surgeries enabling open access to the colon or endoscopic C-OCE variant. Undoubtedly, its realization poses several challenges, such as sufficiently precise positioning of the probe and the issues related to the utilization of reference silicone layers without direct visual control. In this context, it can be mentioned that in principle the endoscopic OCT channel can be combined with a video-camera channel (which is routinely used in ophthalmic OCT systems) to enable visual navigation. An important issue is the use of reference silicone layer in the endoscopic mode. It should be emphasized that the correct mechanical response, i.e., the response determined by the Young’s modulus without an appreciable contribution of the much greater bulk modulus, can be obtained only when the stiction at the interfaces of the reference silicone with the OCT probe and tissue is minimized [see a detailed discussion of this issue in paper (44)]. This means, in particular, that it is impossible to fix the reference silicone layer at the output window of OCT probe by gluing. Our preliminary test suggests that a thin probe cover (similar to that used for ultrasound examinations in gynecology) can be used to hold a piece of the reference silicone at the distal end of the OCT probe with a drop of saline added as a lubricant to minimalize the above-mentioned stiction. The applied level of compression can be then controlled by calculating in real time the strain in the reference silicone layer (36, 71) with the simultaneous accounting for the possible noticeable changes in the silicone thickness (72). The existing ideas about the potential approaches to address these challenges make it possible in the future to hope for the implementation of endoscopic C-OCE for the clinical diagnosis of colon diseases.

To summarize, we demonstrated the possibility of C-OCE to distinguish malignant tumor tissue from benign tumor/non-tumor tissue of colorectal cancer according to qualitative and quantitative characteristics. The high correlation dependence of the results of histological and C-OCE segmentation of cancer cell areas suggests high potential in determining the exact location of the tumor by C-OCE for targeted biopsy. For the first time, the stiffness characteristics of colorectal cancer morphological structures were demonstrated using C-OCE. Based on the analysis of C-OCE images, qualitative and quantitative criteria were established to distinguish tumorous from normal colon tissue, benign adenoma from malignant cancer, low-grade from high-grade CRAC, including identification of specific morphological features in colorectal cancer tissue (cribriform pattern CRAC and extracellular mucin fields MAC). Further and vaster study of other morphological subtypes by C-OCE and the parallel development of endoscopic C-OCE-probe for clinical examination opens up the perspective of the rapidly developing C-OCE method to become a valid diagnostic tool for examining colorectal cancer morphology and performing targeted biopsy of colorectal cancer.

## Data availability statement

The raw data supporting the conclusions of this article will be made available by the authors, without undue reservation.

## Ethics statement

The studies involving human participants were reviewed and approved by Institutional Review Board of the Privolzhsky Research Medical University (REB#3 of February 21, 2020). The patients/participants provided their written informed consent to participate in this study. 

## Author contributions

Conceptualization, AP, NG and MS. Methodology, AP and VZ. Software, VZ and AS. Validation, SK, MK, EK and VZ. Writing, AP, MS, VZ and NG. Visualization, AP, EK and EG. Supervision, EZ, AM, MS, NG and VZ. All authors contributed to the article and approved the submitted version.

## References

[1] Bray F Ferlay J Soerjomataram I Siegel RL Torre LA Jemal A. Global cancer statistics 2018: GLOBOCAN estimates of incidence and mortality worldwide for 36 cancers in 185 countries. Cancer J Clin (2018) 68(6):394–424. doi: 10.3322/caac.21492 30207593

[2] Shih IM Wang TL Traverso G Romans K Hamilton SR Ben-Sasson S. Top-down morphogenesis of colorectal tumors. Proc Natl Acad Sci U.S.A. (2001) 98(5):2640–5. doi: 10.1073/pnas.051629398 PMC30191 11226292

[3] Argilés G Tabernero J Labianca R Hochhauser D Salazar R Iveson T. Localised colon cancer: ESMO clinical practice guidelines for diagnosis, treatment and follow-up. Ann Oncol (2020) 31(10):1291–305. doi: 10.1016/j.annonc.2020.06.022 32702383

[4] Kijima S Sasaki T Nagata K Utano K Lefor AT Sugimoto H. Preoperative evaluation of colorectal cancer using CT colonography, MRI, and PET/CT. World J Gastroenterol (2014) 20(45):16964–75. doi: 10.3748/wjg.v20.i45.16964 PMC4258565 25493009

[5] Kekelidze M D'Errico L Pansini M Tyndall A Hohmann J. Colorectal cancer: current imaging methods and future perspectives for the diagnosis, staging and therapeutic response evaluation. World J Gastroenterol (2013) 19(46):8502–14. doi: 10.3748/wjg.v19.i46.8502 PMC3870495 24379567

[6] Lopes G Stern MC Temin S Sharara AI Cervantes A Costas-Chavarri A. Early detection for colorectal cancer: ASCO resource-stratified guideline. J Global Oncol (2019) 5:1–22. doi: 10.1200/jgo.18.00213 PMC6426543 30802159

[7] Remo A Fassan M Vanoli A Bonetti LR Barresi V Tatangelo F. Morphology and molecular features of rare colorectal carcinoma histotypes. Cancers (2019) 11(7):1036. doi: 10.3390/cancers11071036 31340478 PMC6678907

[8] Shia J Schultz N Kuk D Vakiani E Middha S Segal NH. Morphological characterization of colorectal cancers in the cancer genome atlas reveals distinct morphology-molecular associations: clinical and biological implications. Modern Pathol (2017) 30(4):599–609. doi: 10.1038/modpathol.2016.198 PMC5380525 27982025

[9] Gerlinger M Rowan AJ Horswell S Math M Larkin J Endesfelder D. Intratumor heterogeneity and branched evolution revealed by multiregion sequencing. N Engl J Med (2012) 366(10):883–92. doi: 10.1056/NEJMoa1113205 PMC4878653 22397650

[10] Müller MF Ibrahim AEK Arends MJ. Molecular pathological classification of colorectal cancer. Virchows Archiv an Int J Pathol (2016) 469(2):125–34. doi: 10.1007/s00428-016-1956-3 PMC4978761 27325016

[11] Sveen A Kopetz S Lothe RA. Biomarker-guided therapy for colorectal cancer: strength in complexity. Nat Rev Clin Oncol (2020) 17(1):11–32. doi: 10.1038/s41571-019-0241-1 31289352 PMC7577509

[12] Sagaert X Vanstapel A Verbeek S. Tumor heterogeneity in colorectal cancer: What do we know so far? Pathobiology (2018) 85(1-2):72–84. doi: 10.1159/000486721 29414818

[13] Kim BH Kim JM Kang GH Chang HJ Kang DW Kim JH. Standardized pathology report for colorectal cancer, 2nd edition. J Pathol Transl Med (2020) 54(1):1–19. doi: 10.4132/jptm.2019.09.28 31722452 PMC6986966

[14] Compton CC. Colorectal carcinoma: diagnostic, prognostic, and molecular features. Mod Pathol (2003) 16(4):376–88. doi: 10.1097/01.mp.0000062859.46942.93 12692203

[15] Kuipers EJ Grady WM Lieberman D Seufferlein T Sung JJ Boelens PG. Colorectal cancer. Nat Rev Dis Primers (2015) 1:15065. doi: 10.1038/nrdp.2015.65 27189416 PMC4874655

[16] Díaz Del Arco C Domínguez Serrano I Fernández Aceñero MJ. Colorectal cribriform comedo-type adenocarcinoma: a distinct subtype with poor prognosis? Acta Gastroenterol Belg (2019) 82(2):329–32. 31314198

[17] Taylor AS Liu N Fang JM Panarelli N Zhao L Cheng J. Cribriform colon cancer: a morphological growth pattern associated with extramural venous invasion, nodal metastases and microsatellite stability. J Clin Pathol (2022) 75(7):483–7. doi: 10.1136/jclinpath-2021-207485 33782192

[18] Yamada S Osakabe M Eizuka M Hashimoto M Uesugi N Yanagawa N. Cribriform-type adenocarcinoma of the colorectum: comprehensive molecular analyses of a distinctive histologic subtype of colorectal cancer. Carcinogenesis (2022) 43(6):601–10. doi: 10.1093/carcin/bgac029 PMC9234757 35278309

[19] Ahadi M Sokolova A Brown I Chou A Gill AJ. The 2019 world health organization classification of appendiceal, colorectal and anal canal tumours: an update and critical assessment. Pathology (2021) 53(4):454–61. doi: 10.1016/j.pathol.2020.10.010 33461799

[20] Chung CK Zaino RJ Stryker JA. Colorectal carcinoma: evaluation of histologic grade and factors influencing prognosis. J Surg Oncol (1982) 21(3):143–8. doi: 10.1002/jso.2930210302 7132366

[21] Derwinger K Kodeda K Bexe-Lindskog E Taflin H. Tumour differentiation grade is associated with TNM staging and the risk of node metastasis in colorectal cancer. Acta Oncol (2010) 49(1):57–62. doi: 10.3109/02841860903334411 20001500

[22] Vasile L Olaru A Munteanu M Pleşea IE Surlin V Tudoraşcu C. Prognosis of colorectal cancer: clinical, pathological and therapeutic correlation. Rom J Morphol Embryol (2012) 53(2):383–91. 22732811

[23] Oñate-Ocaña LF Montesdeoca R López-Graniel CM Aiello-Crocifoglio V Mondragón-Sánchez R Cortina-Borja M. Identification of patients with high-risk lymph node-negative colorectal cancer and potential benefit from adjuvant chemotherapy. Jpn J Clin Oncol (2004) 34(6):323–8. doi: 10.1093/jjco/hyh054 15333684

[24] Sauer R Becker H Hohenberger W Rödel C Wittekind C Fietkau R. Preoperative versus postoperative chemoradiotherapy for rectal cancer. N Engl J Med (2004) 351(17):1731–40. doi: 10.1056/NEJMoa040694 15496622

[25] Canda AE Terzi C Gorken IB Oztop I Sokmen S Fuzun M. Effects of preoperative chemoradiotherapy on anal sphincter functions and quality of life in rectal cancer patients. Int J Colorectal Dis (2010) 25(2):197–204. doi: 10.1007/s00384-009-0807-y 19784660

[26] Birgisson H Påhlman L Gunnarsson U Glimelius B. Occurrence of second cancers in patients treated with radiotherapy for rectal cancer. J Clin Oncol (2005) 23(25):6126–31. doi: 10.1200/jco.2005.02.543 16135478

[27] Gubarkova EV Sovetsky AA Zaitsev VY Matveyev AL Vorontsov DA Sirotkina MA. OCT-elastography-based optical biopsy for breast cancer delineation and express assessment of morphological/molecular subtypes. Biomed Opt Express (2019) 10(5):2244–63. doi: 10.1364/BOE.10.002244 PMC6524573 31143491

[28] Plekhanov AA Sirotkina MA Sovetsky AA Gubarkova EV Kuznetsov SS Matveyev AL. Histological validation of in vivo assessment of cancer tissue inhomogeneity and automated morphological segmentation enabled by optical coherence elastography. Sci Rep (2020) 10(11781):1–16. doi: 10.1038/s41598-020-68631-w 32678175 PMC7366713

[29] Yashin KS Kiseleva EB Moiseev AA Kuznetsov SS Timofeeva LB Pavlova NP. Quantitative nontumorous and tumorous human brain tissue assessment using microstructural co- and cross-polarized optical coherence tomography. Sci Rep (2019) 9(1):2024. doi: 10.1038/s41598-019-38493-y 30765763 PMC6375924

[30] Zuccaro G Gladkova N Vargo J Feldchtein F Zagaynova E Conwell D. Optical coherence tomography of the esophagus and proximal stomach in health and disease. Am J Gastroenterol (2001) 96(9):2633–9. doi: 10.1111/j.1572-0241.2001.04119.x 11569687

[31] Zagaynova E Gladkova N Shakhova N Gelikonov G Gelikonov V. Endoscopic OCT with forward-looking probe: clinical studies in urology and gastroenterology. J Biophoton (2008) 1(2):114–28. doi: 10.1002/jbio.200710017 19343643

[32] World Medical Association. Ethical principles for medical research involving human subjects. Eur J Emerg Med (2001) 8(3):221–3. doi: 10.1097/00063110-200109000-00010 11587468

[33] Fedyanin MY Gladkov OA Gordeev SS Karachun AM Kozlov NA Mamedli ZZ. Practical guidelines for drug treatment of colon, rectosigmoid junction, and rectal cancer. Malignant Tumors (2022) 12(3s2):401–54. doi: 10.18027/2224-5057-2022-12-3s2-401-454

[34] Nagtegaal ID Odze RD Klimstra D Paradis V Rugge M Schirmacher P. The 2019 WHO classification of tumours of the digestive system. Histopathology (2020) 76(2):182–8. doi: 10.1111/his.13975 PMC7003895 31433515

[35] Lino-Silva LS Salcedo-Hernández RA Herrera-Gómez A Padilla-Rosciano A Ramírez-Jaramillo M Herrera-Goepfert RE. Colonic cribriform carcinoma, a morphologic pattern associated with low survival. Int J Surg Pathol (2015) 23(1):13–9. doi: 10.1177/1066896914542125 25015669

[36] Sovetsky AA Matveyev AL Matveev LA Gubarkova EV Plekhanov AA Sirotkina MA. Full-optical method of local stress standardization to exclude nonlinearity-related ambiguity of elasticity estimation in compressional optical coherence elastography. Laser Phys Lett (2020) 17(6):065601. doi: 10.1088/1612-202x/ab8794

[37] Gelikonov VM Romashov VN Shabanov DV Ksenofontov SY Terpelov DA Shilyagin PA. Cross-polarization optical coherence tomography with active maintenance of the circular polarization of a sounding wave in a common path system. Radiophys Quantum Electron (2018) 60(11):897–911. doi: 10.1007/s11141-018-9856-9

[38] Zaitsev VY Matveyev AL Matveev LA Gelikonov GV Gelikonov VM Vitkin A. Deformation-induced speckle-pattern evolution and feasibility of correlational speckle tracking in optical coherence elastography. J Biomed Optics (2015) 20(7):75006. doi: 10.1117/1.jbo.20.7.075006 26172612

[39] Zaitsev VY Matveyev AL Matveev LA Sovetsky AA Hepburn MS Mowla A. Strain and elasticity imaging in compression optical coherence elastography: The two-decade perspective and recent advances. J Biophoton (2021) 14(2):e202000257. doi: 10.1002/jbio.202000257 32749033

[40] Zaitsev VY Matveyev AL Matveev LA Gelikonov GV Sovetsky AA Vitkin A. Optimized phase gradient measurements and phase-amplitude interplay in optical coherence elastography. J Biomed Optics (2016) 21(11):116005. doi: 10.1117/1.JBO.21.11.116005 27824215

[41] Matveyev AL Matveev LA Sovetsky AA Gelikonov GV Moiseev AA Zaitsev VY. Vector method for strain estimation in phase-sensitive optical coherence elastography. Laser Phys Lett (2018) 15(6):065603. doi: 10.1088/1612-202x/aab5e9

[42] Sovetsky AA Matveyev AL Matveev LA Shabanov DV Zaitsev VY. Manually-operated compressional optical coherence elastography with effective aperiodic averaging: demonstrations for corneal and cartilaginous tissues. Laser Phys Lett (2018) 15:085602. doi: 10.1088/1612-202X/aac879

[43] Kennedy KM Chin L McLaughlin RA Latham B Saunders CM Sampson DD. Quantitative micro-elastography: imaging of tissue elasticity using compression optical coherence elastography. Sci Rep (2015) 5:15538. doi: 10.1038/srep15538 26503225 PMC4622092

[44] Zaitsev VY Matveyev AL Matveev LA Gubarkova EV Sovetsky AA Sirotkina MA. Practical obstacles and their mitigation strategies in compressional optical coherence elastography of biological tissues. J Innov Opt Health Sci (2017) 10(06):1742006. doi: 10.1142/S1793545817420068

[45] Plekhanov AA Gubarkova EV Sovietsky AA Zaitsev VY Matveev LA Matveyev AL. Optical coherence elastography for non-invasive monitoring of tumor elasticity under chemotherapy: Pilot study. Sovremennye Tehnol v Med (2018) 10(3):9. doi: 10.17691/stm2018.10.3.5

[46] Gubarkova EV Sovetsky AA Matveev LA Matveyev AL Vorontsov DA Plekhanov AA. Nonlinear elasticity assessment with optical coherence elastography for high-selectivity differentiation of breast cancer tissues. Materials (2022) 15(9):3308. doi: 10.3390/ma15093308 35591642 PMC9099511

[47] Gubarkova EV Sovetsky AA Vorontsov DA Buday PA Sirotkina MA Plekhanov AA. Compression optical coherence elastography versus strain ultrasound elastography for breast cancer detection and differentiation: pilot study. Biomed Opt Express (2022) 13(5):2859–81. doi: 10.1364/boe.451059 PMC9203088 35774307

[48] Giavarina D. Understanding bland altman analysis. Biochem Med (2015) 25(2):141–51. doi: 10.11613/bm.2015.015 PMC4470095 26110027

[49] Wang C Zhang Q Wu X Tang T Liu H Zhu SW. Quantitative diagnosis of colorectal polyps by spectral domain optical coherence tomography. BioMed Res Int (2014) 2014:570629. doi: 10.1155/2014/570629 PMC4000955 24818145

[50] Ding Q Deng Y Yu X Yuan J Zeng Z Mu G. Rapid, high-resolution, label-free, and 3-dimensional imaging to differentiate colorectal adenomas and non-neoplastic polyps with micro-optical coherence tomography. Clin Trans Gastroenterol (2019) 10(6):e00049. doi: 10.14309/ctg.0000000000000049 PMC6613865 31192828

[51] Zhao Q Zhou C Wei H He Y Chai X Ren Q. Ex vivo determination of glucose permeability and optical attenuation coefficient in normal and adenomatous human colon tissues using spectral domain optical coherence tomography. J Biomed Optics (2012) 17(10):105004. doi: 10.1117/1.JBO.17.10.105004 23223998

[52] Ashok PC Praveen BB Bellini N Riches A Dholakia K Herrington CS. Multi-modal approach using raman spectroscopy and optical coherence tomography for the discrimination of colonic adenocarcinoma from normal colon. Biomed Opt Express (2013) 4(10):2179–86. doi: 10.1364/BOE.4.002179 PMC3799675 24156073

[53] Yi J Radosevich AJ Stypula-Cyrus Y Mutyal NN Azarin SM Horcher E. Spatially resolved optical and ultrastructural properties of colorectal and pancreatic field carcinogenesis observed by inverse spectroscopic optical coherence tomography. J Biomed Optics (2014) 19(3):36013. doi: 10.1117/1.JBO.19.3.036013 PMC4019430 24643530

[54] Zeng Y Chapman WC Jr. Lin Y Li S Mutch M Zhu Q. Diagnosing colorectal abnormalities using scattering coefficient maps acquired from optical coherence tomography. J Biophoton (2021) 14(1):e202000276. doi: 10.1002/jbio.202000276 PMC8196414 33064368

[55] Li Y Zhu Z Chen JJ Jing JC Sun CH Kim S. Multimodal endoscopy for colorectal cancer detection by optical coherence tomography and near-infrared fluorescence imaging. Biomed Opt Express (2019) 10(5):2419–29. doi: 10.1364/boe.10.002419 PMC6524571 31143497

[56] Adler DC Zhou C Tsai T-H Schmitt J Huang Q Mashimo H. Three-dimensional endomicroscopy of the human colon using optical coherence tomography. Opt Express (2009) 17(2):784–96. doi: 10.1364/oe.17.000784 PMC2886288 19158891

[57] Zeng Y Xu S Chapman WC Jr. Li S Alipour Z Abdelal H. Real-time colorectal cancer diagnosis using PR-OCT with deep learning. Theranostics (2020) 10(6):2587–96. doi: 10.7150/thno.40099 PMC7052898 32194821

[58] Saratxaga CL Bote J Ortega-Morán JF Picón A Terradillos E del Río NA. Characterization of optical coherence tomography images for colon lesion differentiation under deep learning. Appl Sci (2021) 11(7):3119. doi: 10.3390/app11073119

[59] Luo H Li S Zeng Y Cheema H Otegbeye E Ahmed S. Human colorectal cancer tissue assessment using optical coherence tomography catheter and deep learning. J Biophoton (2022) 15(6):e202100349. doi: 10.1002/jbio.202100349 PMC9581715 35150067

[60] Amygdalos I Hachgenei E Burkl L Vargas D Goßmann P Wolff LI. Optical coherence tomography and convolutional neural networks can differentiate colorectal liver metastases from liver parenchyma ex vivo. J Cancer Res Clin Oncol (2022). doi: 10.1007/s00432-022-04263-z PMC10314842 35960377

[61] Cârţână ET Gheonea DI Săftoiu A. Advances in endoscopic ultrasound imaging of colorectal diseases. World J Gastroenterol (2016) 22(5):1756–66. doi: 10.3748/wjg.v22.i5.1756 PMC4724607 26855535

[62] Uçar A Obuz F Sökmen S Terzi C Sağol O Sarıoğlu S. Efficacy of high resolution magnetic resonance imaging in preoperative local staging of rectal cancer. Mol Imaging Radionucl Ther (2013) 22(2):42–8. doi: 10.4274/Mirt.43153 PMC3759308 24003396

[63] Maas M Lambregts DMJ Nelemans PJ Heijnen LA Martens MH Leijtens JWA. Assessment of clinical complete response after chemoradiation for rectal cancer with digital rectal examination, endoscopy, and MRI: Selection for organ-saving treatment. Ann Surg Oncol (2015) 22(12):3873–80. doi: 10.1245/s10434-015-4687-9 PMC4595525 26198074

[64] Han C Tang X Yang M Zhang K Liu J Lin R. How useful is endoscopic ultrasound in differentiating T3/T4a T stage of colorectal cancer: A prospective study. Front Oncol (2021) 11:618512. doi: 10.3389/fonc.2021.618512 35127462 PMC8813747

[65] Tsung PC Park JH Kim YS Kim SY Park WW Kim HT. Miniprobe endoscopic ultrasonography has limitations in determining the T stage in early colorectal cancer. Gut Liver (2013) 7(2):163–8. doi: 10.5009/gnl.2013.7.2.163 PMC3607769 23560151

[66] Eckardt AJ Jenssen C. Current endoscopic ultrasound-guided approach to incidental subepithelial lesions: optimal or optional? Ann Gastroenterol (2015) 28(2):160–72. PMC4367205 25830949

[67] Saftoiu A Vilman P. Endoscopic ultrasound elastography– a new imaging technique for the visualization of tissue elasticity distribution. J Gastrointest Liver Dis JGLD (2006) 15(2):161–5. 16802011

[68] Waage JE Havre RF Odegaard S Leh S Eide GE Baatrup G. Endorectal elastography in the evaluation of rectal tumours. Colorectal Dis Off J Assoc Coloproctol Great Britain Ireland (2011) 13(10):1130–7. doi: 10.1111/j.1463-1318.2010.02440.x 21040360

[69] Waage JE Bach SP Pfeffer F Leh S Havre RF Ødegaard S. Combined endorectal ultrasonography and strain elastography for the staging of early rectal cancer. Colorectal Dis Off J Assoc Coloproctol Great Britain Ireland (2015) 17(1):50–6. doi: 10.1111/codi.12764 25176033

[70] Esaki M Yamamura T Nakamura M Maeda K Sawada T Mizutani Y. Endoscopic ultrasound elastography as a novel diagnostic method for the assessment of hardness and depth of invasion in colorectal neoplasms. Digestion (2021) 102(5):701–13. doi: 10.1159/000511589 33207360

[71] Zaitsev VY Ksenofontov SY Sovetsky AA Matveyev AL Matveev LA Zykov AA. Real-time strain and elasticity imaging in phase-sensitive optical coherence elastography using a computationally efficient realization of the vector method. Photonics (2021) 8(12):527. doi: 10.3390/photonics8120527

[72] Sovetsky AA Matveyev AL Matveev LA Gelikonov GV Zaitsev VY. Mapping Large strains in phase-sensitive OCT: Key role of supra-pixel displacement tracking in incremental strain evaluation. J Biomed Photon Eng (2022) 8(3):30304. doi: 10.18287/jbpe22.08.030304

